# Supplementary material for: The widespread increase in inter-individual variability of gene expression in the human brain with age
Source: Aging (Albany NY). 2019 Apr 19;11(8):2253–80. doi: 10.18632/aging.101912 (PMC6520006; doi:10.18632/aging.101912)
Supplement: Supplemental File [file aging-11-101912-s001.pdf]

SUPPLEMENTARY MATERIAL

Please browse the links in Full Text version of this manuscript to see Supplementary Tables 1-7.

Supplemental Table 1. The results of the differential expression analysis performed on regression-corrected and SVA-corrected data.

Supplemental Table 2. The results of the GSEA Analysis for BP GO categories performed on the differentially expressed genes obtained on regression-corrected and SVA-corrected data.

Supplemental Table 3. The results of the differential variability analysis while applying "continuous approach" for the regression and SVA-corrected expression data.

Supplemental Table 4. The results of the differential variability analysis while applying "grouped approach" for the regression and SVA-corrected expression data.

Supplemental Table 5. The results of GSEA Analysis among the KEGG pathways on the differential variability measures from the "continuous" and "grouped" approaches.

Supplemental Table 6. The results of GSEA Analysis among the GO categories on the differential variability measures from the "continuous" and "grouped" approaches.

Supplemental Table 7. The median variability measures for the genes within each KEGG pathway and GO term, using different approaches.

Supplemental Information

Post-mortem interval associated differentially variable genes

Recent reports made on the GTEx data [1] suggest that extent of Post-mortem interval (PMI) – the time passed between death and sample collection, could be associated with the degradation of specific mRNA species and, consequently, change in both mean and variability of their gene expression. We have checked whether our set of differentially variable genes (DV) contains any of the reported 266 genes that are susceptible to the degradation dependent on PMI. We found 14, 14, 10 and 7 of those genes among the set of differentially variable genes identified in the grouped approach with quantile normalization, regression, ComBat and SVA correction, respectively.

Overlap of the DV genes with other publications

The total set of differentially variable genes identified by Brinkmeyer-Langford [2] comprises 848 distinct genes found across 13 brain regions. 171 of those genes are specific to the cortex and frontal cortex. Our set of DV genes found in grouped approach after regression correction (N total = 741) has an overlap of 21 genes with Brinkmeyer-Langford and 19 genes for the SVA-corrected expression (N total = 746). The percentage of overlap doesn't increase, if we only consider cortex-specific DV genes from Brinkmeyer-Langford - it constitutes 4 and 3 genes for regression and SVA corrected sets, respectively.

Supplemental References

1. Zhu Y, Wang L, Yin Y, Yang E. Systematic analysis of gene expression patterns associated with postmortem interval in human tissues. Sci Rep. 2017; 7:5435. https://doi.org/10.1038/s41598-017-05882-0

2. Brinkmeyer-Langford CL, Guan J, Ji G, Cai JJ. Aging Shapes the Population-Mean and -Dispersion of Gene Expression in Human Brains. Front Aging Neurosci. 2016; 8:183. https://doi.org/10.3389/fnagi.2016.00183.

Supplemental Table 8. Percentage of KEGG pathways and GO terms that have median expression variability above 0, according to different approaches.

	**Continuous approach**				**Grouped approach**			
	Regression	SVA	QN	ComBat	Regression	SVA	QN	ComBat
**KEGG pathways**	94%	88%	99%	88%	77%	74%	94%	95%
**GO terms**	92%	84%	98%	79%	66%	61%	88%	88%

Supplemental Figures

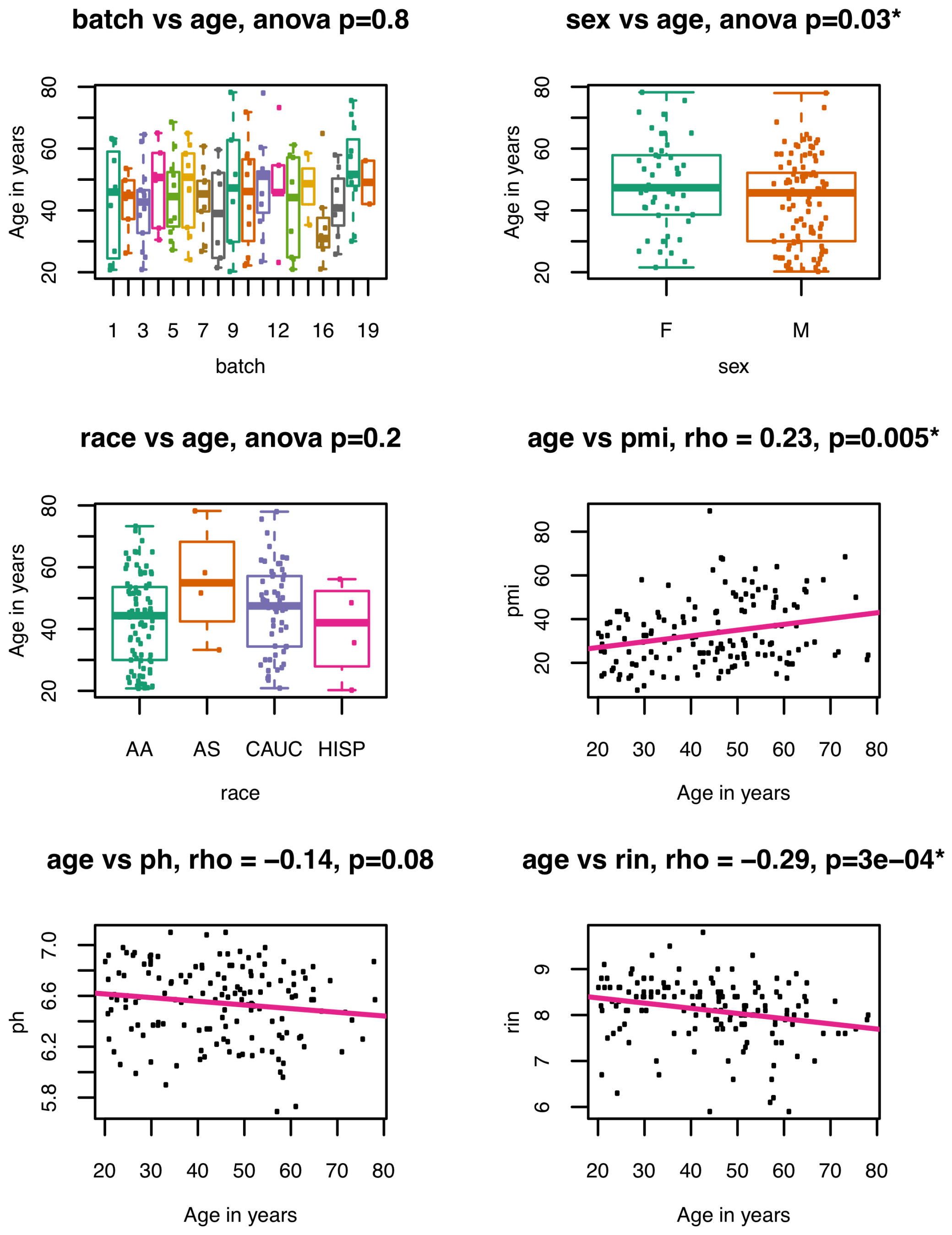

Supplemental Figure 1. Dependency plots between the age and different biological and technical factors, such as batch, sex, race, post-mortem interval (PMI), pH and RNA integrity number (RIN). Box plots were constructed for categorical covariates (batch, sex and race), while scatter plots were made for the numerical covariates (PMI, pH, RIN). In order to test for the significance of the dependence between categorical covariates and age ANOVA-test was performed (p value is displayed on the graph), Spearman correlation estimate was calculated to estimate relationship between numerical covariates and age (Spearman rho and p value are displayed on the graph). * $p \leq 0.05$

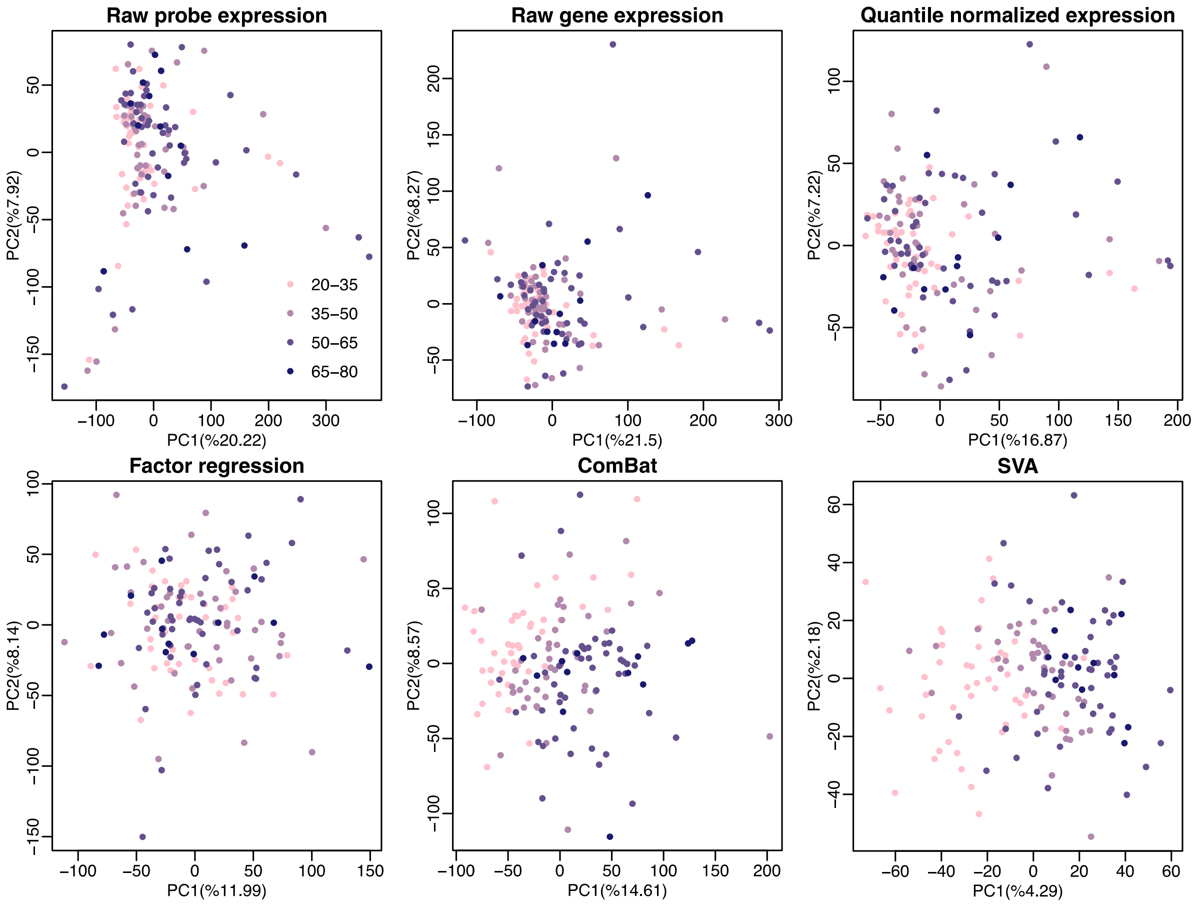

Supplemental Figure 2. PCA plots of the raw expression data and after different pre-processing steps. First row from left to right: probe expression level, gene expression level and quantile normalized gene expression level. Second row from left to right: quantile normalized data further corrected either with regression, ComBat or SVA. Color gradient depicts age range from pink – young to violet – old.

A

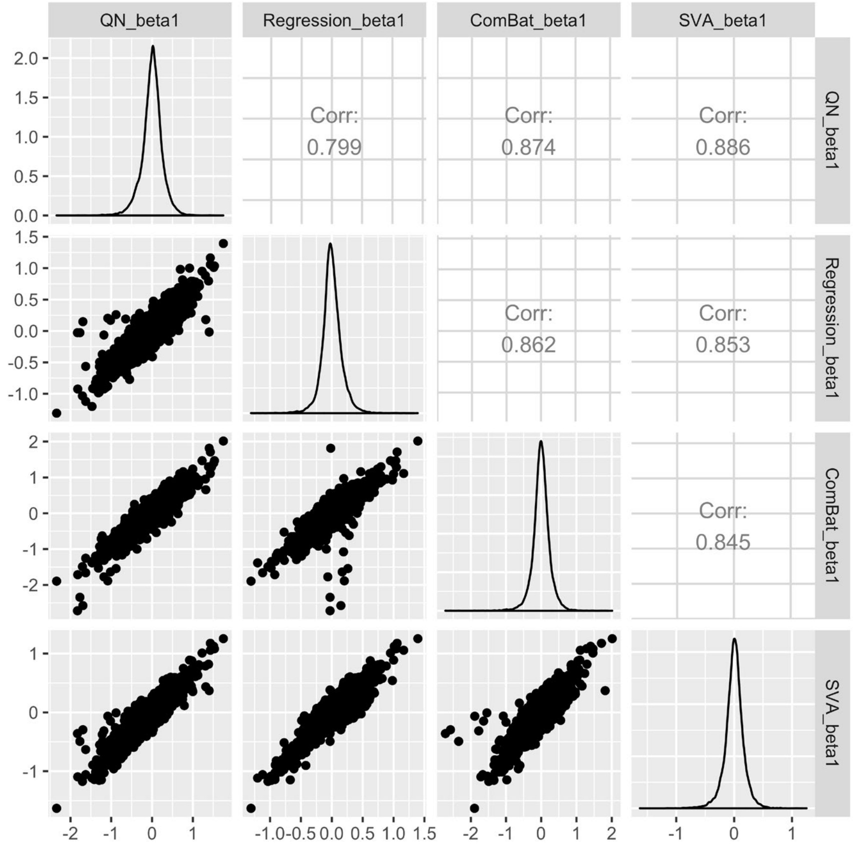

B

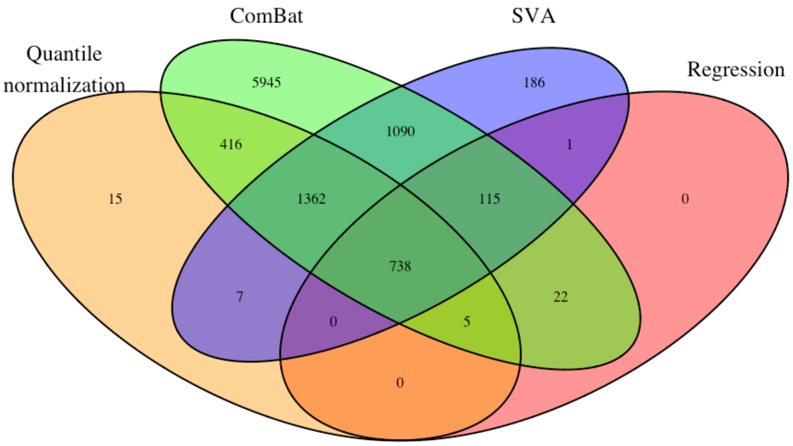

Supplemental Figure 3. (**A**) Illustration of the relationship between beta1 – coefficients from the linear regression models that explain gene expression corrected with different approaches (QN only, QN followed by regression, ComBat, SVA) using age$^{0.25}$. Scatter plots that illustrate relationship between the beta1 – coefficients from different corrections are displayed in the lower triangle of the matrix, distributions of the beta1 – coefficients are located on the diagonal and Spearman correlation estimates are displayed in the upper triangle of the matrix. (**B**) Venn diagram of the differentially expressed (DE) genes with age that were identified after applying different expression correction approaches (QN only, QN followed by regression, ComBat, SVA). The ComBat approach seem to greatly outnumber all the other approaches in the number of DE genes. We consider that consecutive application (6 times) of the linear model that contains age and particular confounding factor caused an overfitting of gene expression with age and lead to identification of so many DE genes, many of which are false positives.

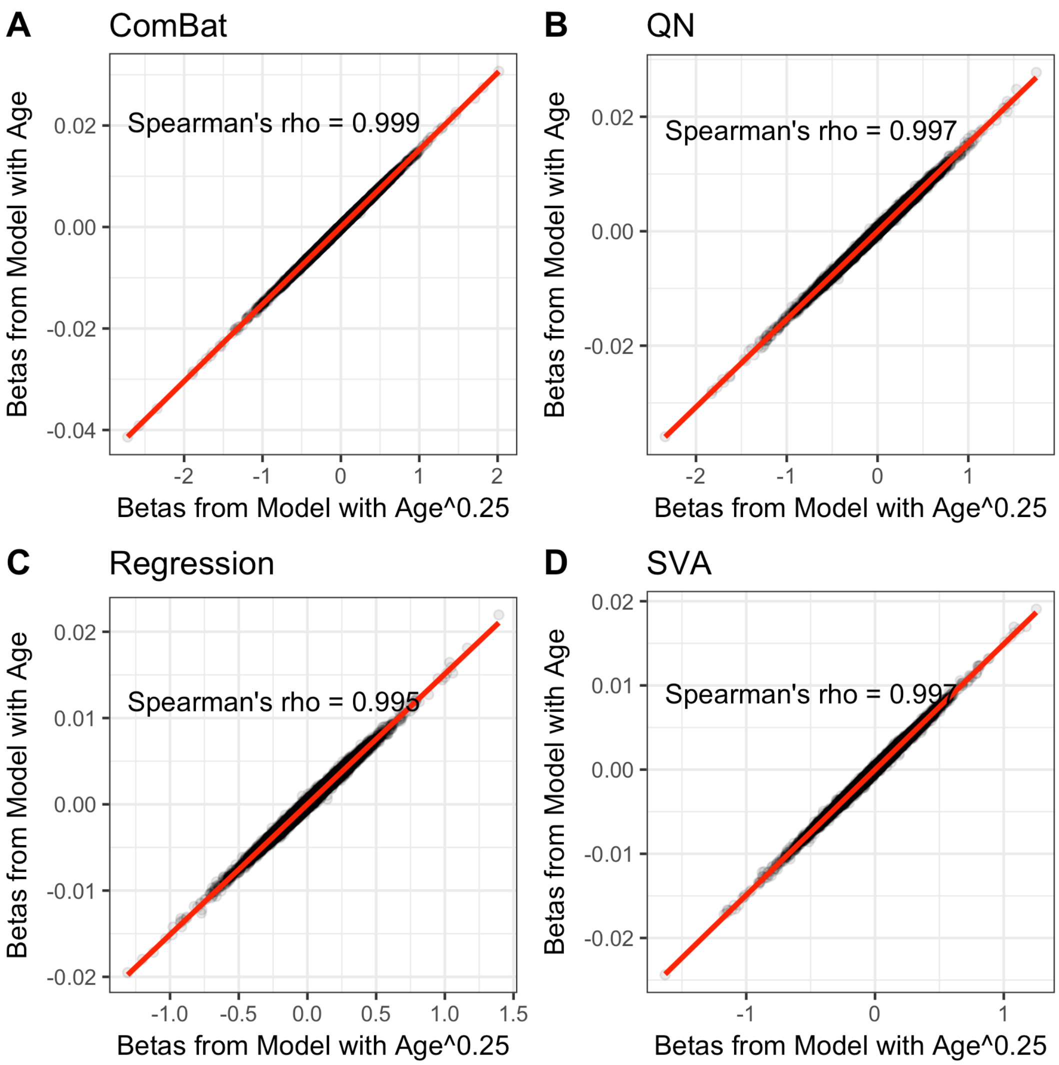

Supplemental Figure 4. Scatter plots of the dependency between beta1 coefficients from the linear regression models that explain gene expression using age in comparison to age$^{0.25}$. Gene expression was corrected using ComBat (**A**), QN only (**B**), regression (**C**) and SVA (**D**). Linear regression line between the variables (in red) and Spearman correlation estimate is shown on the graph.

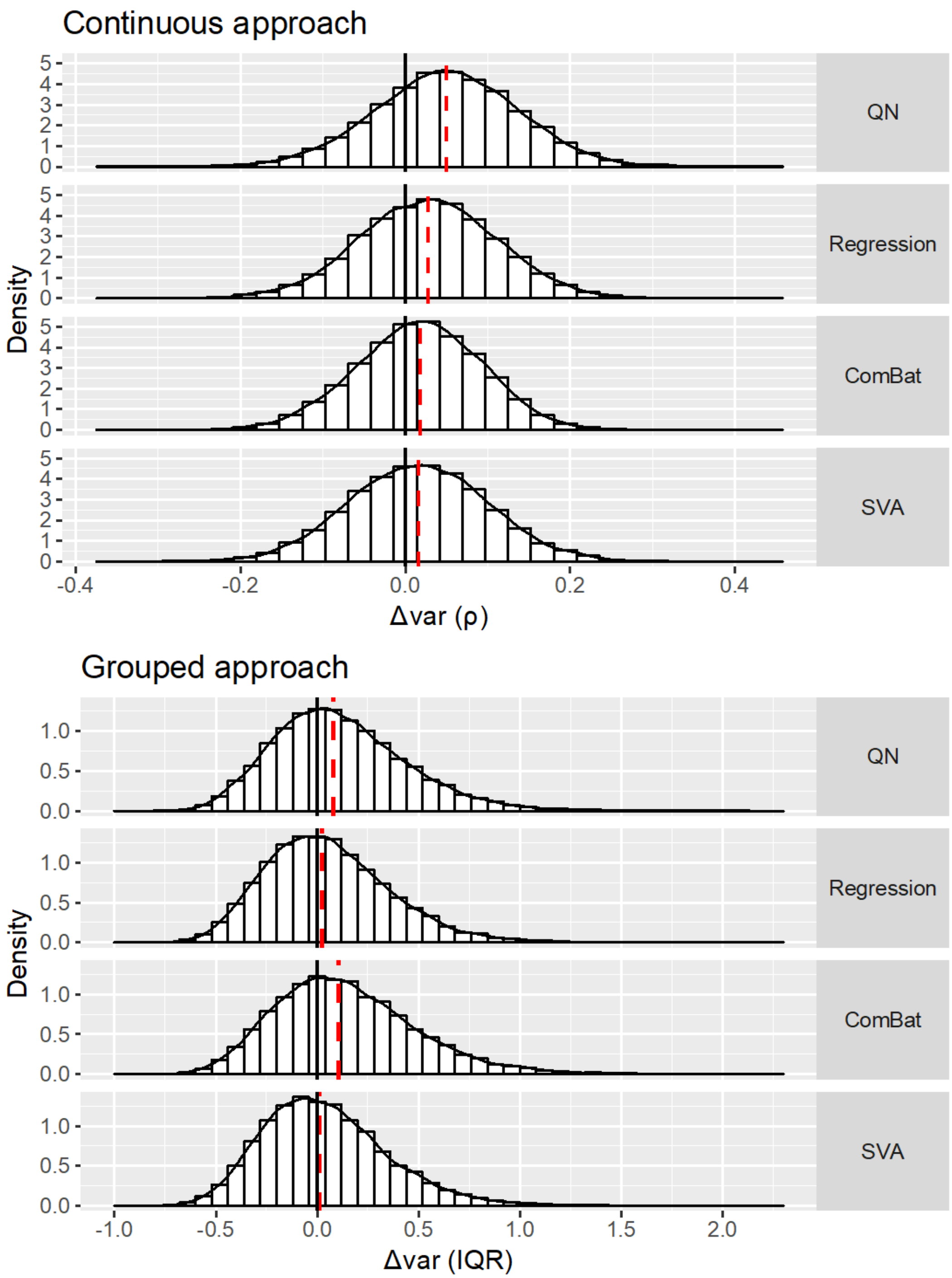

Supplemental Figure 5. Comparison of the distributions of the differential variability measures, Δvar (ρ) and Δvar (IQR), obtained in the expression data corrected with QN, Regression, SVA and ComBat. The black straight line depicts zero, the red dashed line – median of the distribution.

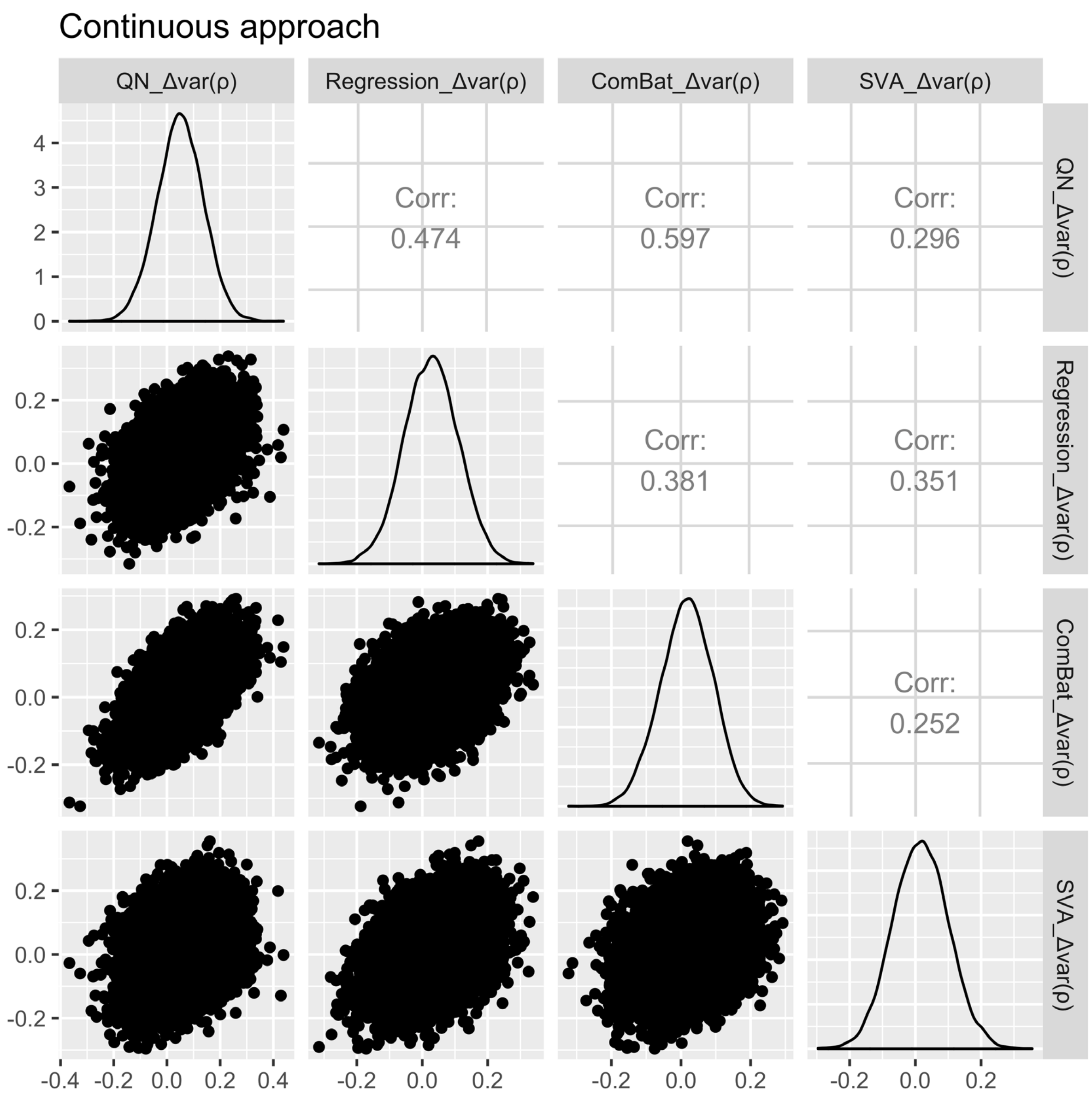

Supplemental Figure 6. Combination of comparisons of the variability measures (Δvar) obtained in the continuous approach between different ways of data pre-processing. Correction with quantile normalization only, linear regression, SVA and ComBat. Scatter plots between the differential variability measures obtained after different corrections are displayed in the lower triangle of the matrix, distributions of the differential variability measures are located on the diagonal and Spearman correlation estimates between the variability measures from different corrections are shown in the upper triangle of the matrix.

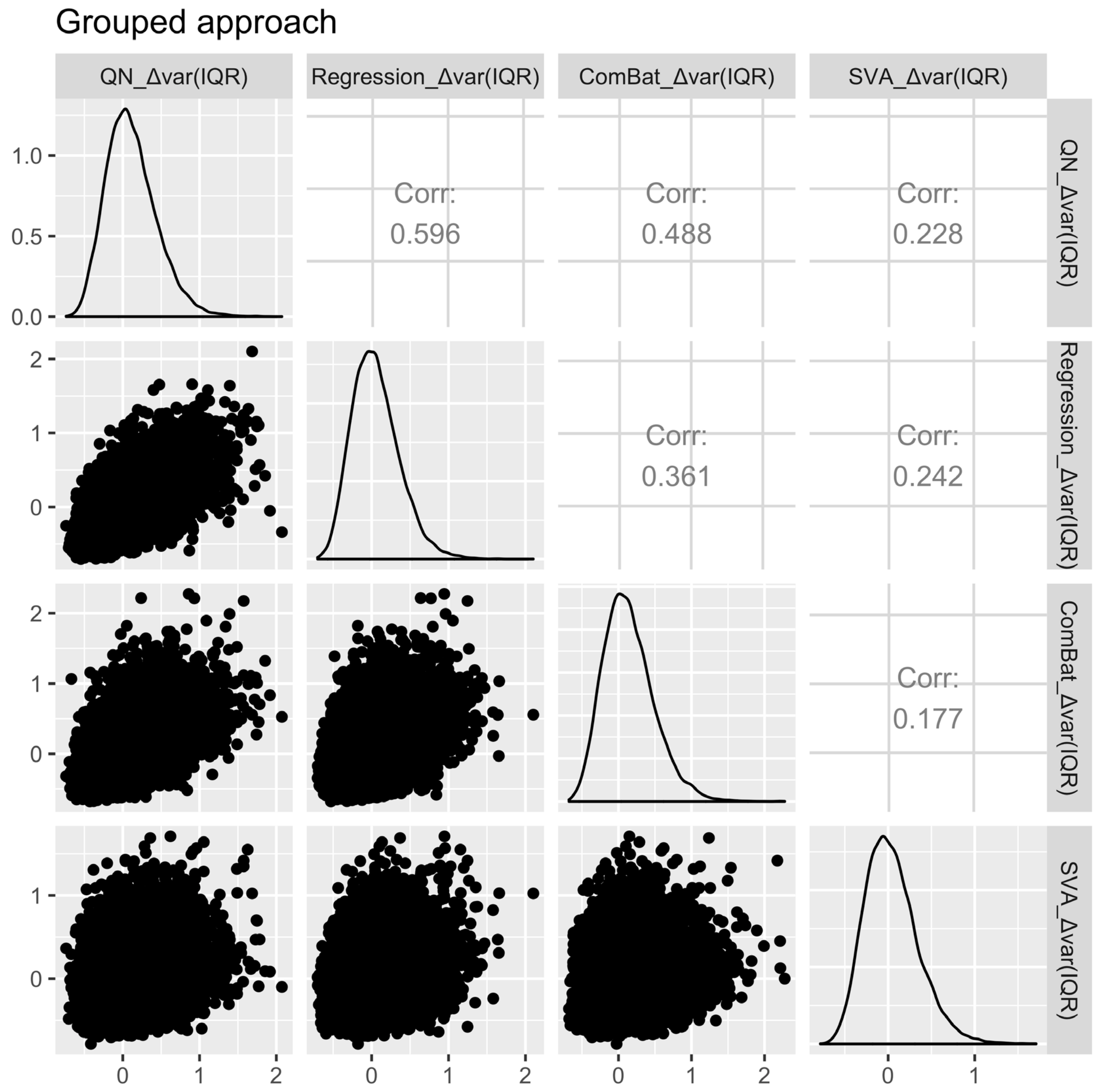

Supplemental Figure 7. Combination of comparisons of the variability measures (Δvar) obtained in the grouped approach between different ways of data pre-processing. Correction with quantile normalization only, linear regression, SVA and ComBat. Scatter plots between the differential variability measures obtained after different corrections are displayed in the lower triangle of the matrix, distributions of the differential variability measures are located on the diagonal and Spearman correlation estimates between the differential variability measures from different corrections are shown in the upper triangle of the matrix.

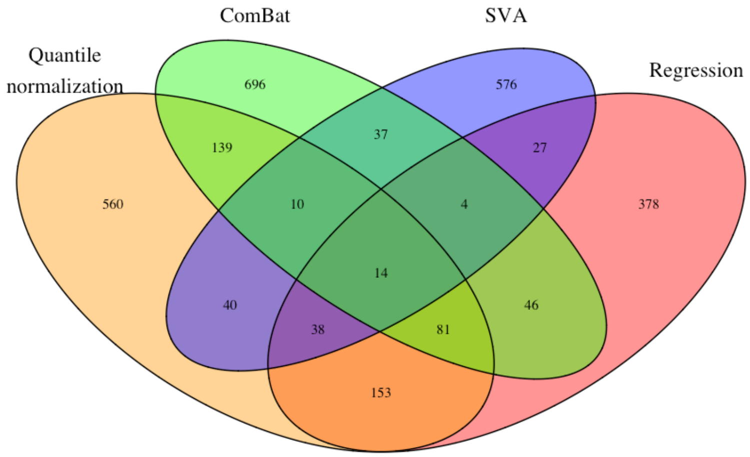

Supplemental Figure 8. Venn diagram of the differentially variable genes found in the grouped approach when expression data was corrected with QN, linear regression, SVA and ComBat.

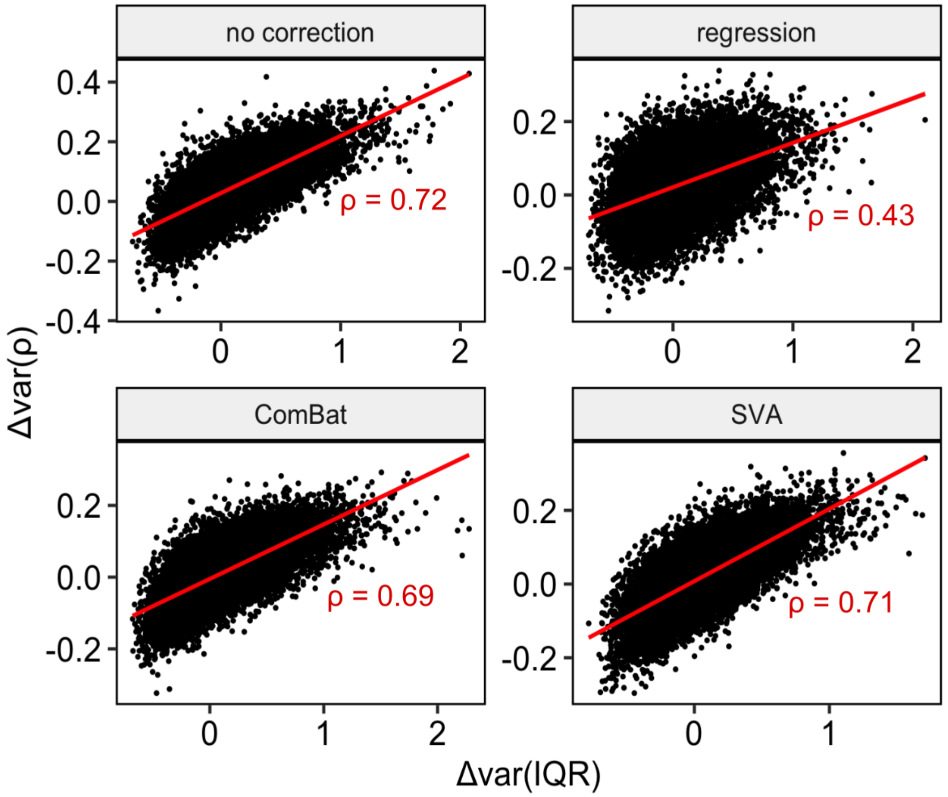

Supplemental Figure 9. Scatter plots of the relationship between the change in the variability measures for the continuous and grouped approaches in data, corrected with QN, ComBat, regression and SVA corrections. Linear regression line between the variables (in red) and Spearman correlation estimate are shown on the graph.

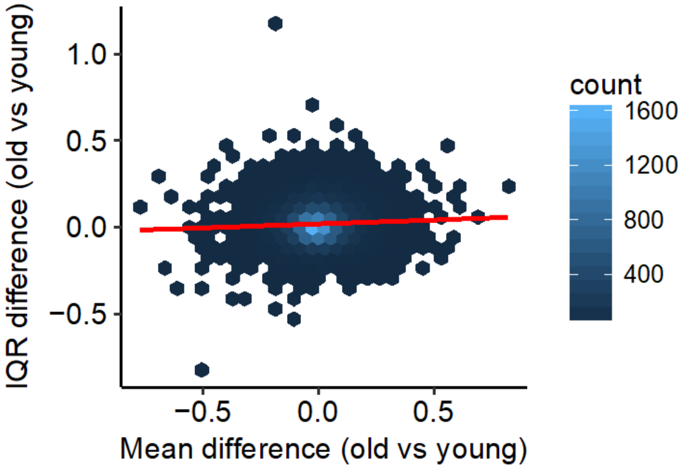

Supplemental Figure 10. Relationship between the change in gene expression level and variability. x axis depicts difference between mean of the old group and mean of the young group distribution (generated by resampling young group with sample size of 22 for 10,000 times and calculating the mean), y axis illustrates difference between interquartile range (IQR) for the old and mean IQR from the young distribution (generated by resampling young group with sample size of 22 for 10,000 times and calculating IQR).

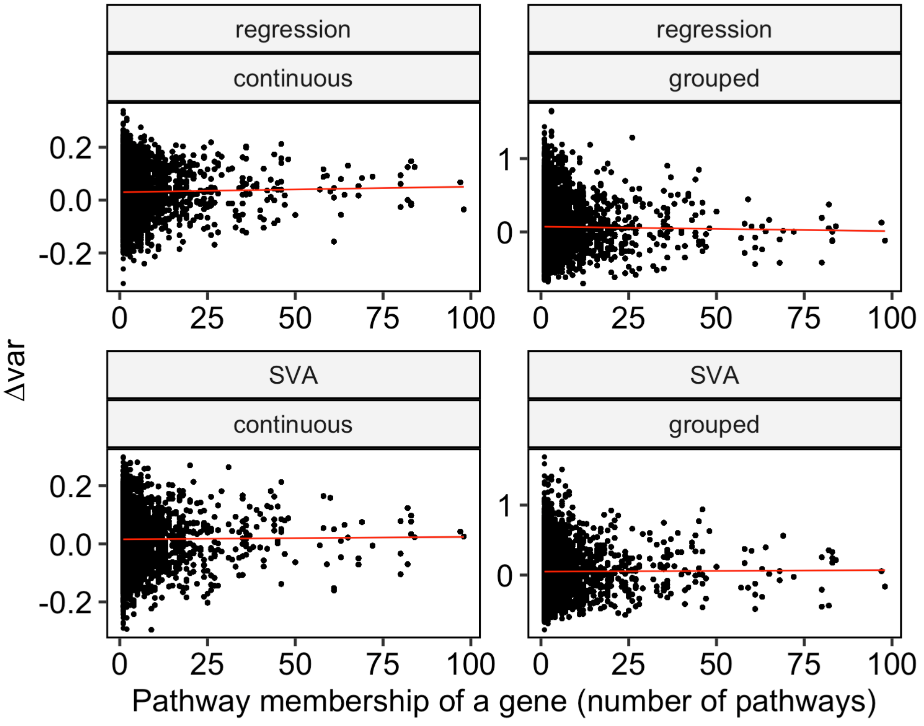

Supplemental Figure 11. Scatter plots that illustrate relationship between the pathway membership of a gene and its change in variability measure for the continuous and grouped approaches in data corrected with regression and SVA. Linear regression line between the variables is shown red on the graph.

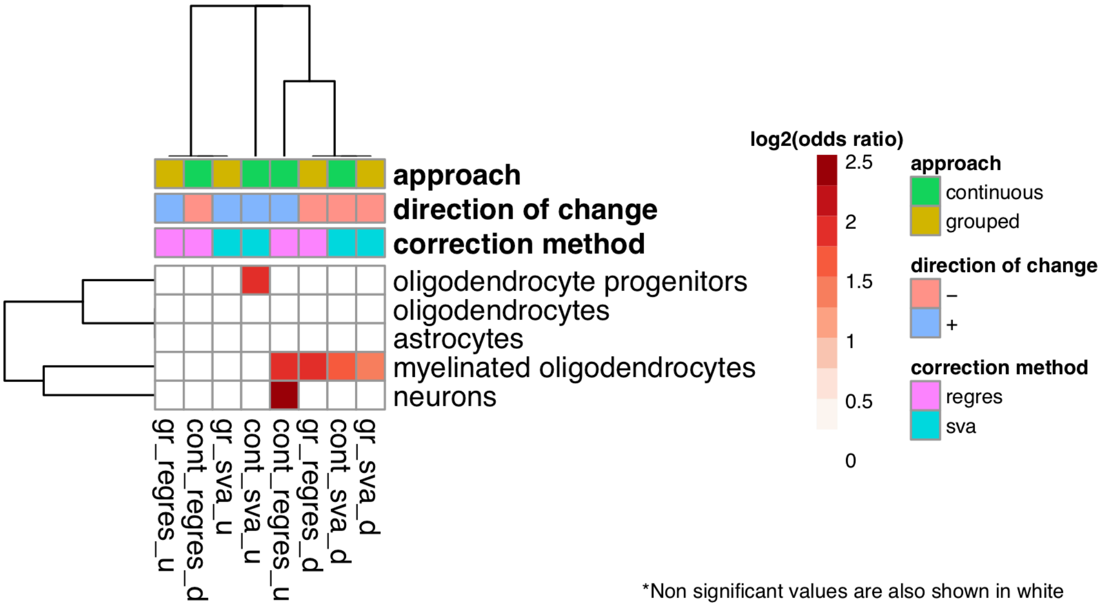

Supplemental Figure 12. A heatmap showing the significant association between differentially variable and cell-type specific genes. Rows show different cell-types and columns show genes detected as differentially variable based on different approaches (grouped (gr_) or continuous (cont_)), correction methods (regression (regres_) or SVA) and direction of change (increase (u) or decrease (d)). The color shows log2(odds ratio) for the enrichment of a particular category in cell-type specific genes. All non-significant ($p > 0.05$) values are discarded and shown as white. Since there are no significantly differentially variable gene detected based on continuous approach, we took the first 1% of genes with the highest change in variability as the DV genes for this analysis. The number of genes tested for each cell type were as follows: oligodendrocyte progenitors 396, oligodendrocytes 64, astrocytes 21, myelinated oligodendrocytes 448, neurons 340.

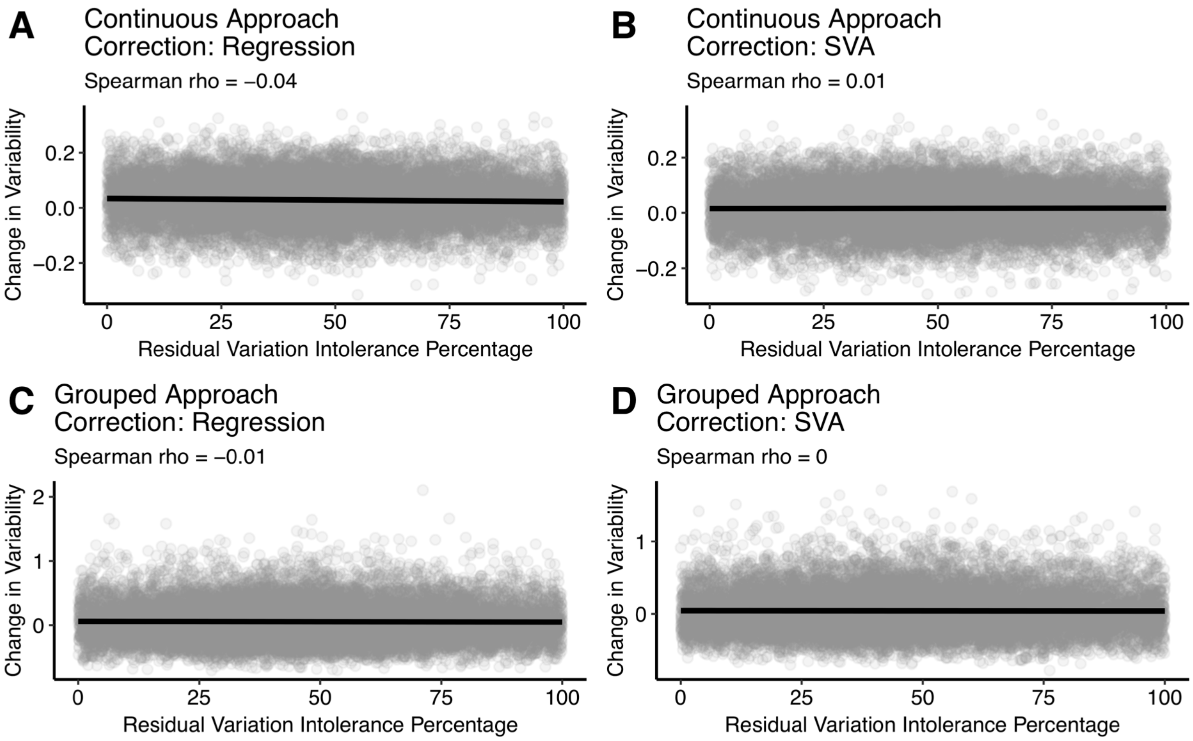

Supplemental Figure 13. Scatter plot showing the association between the change in variability with age (y-axis) and residual variation intolerance (RVI) percentage (x-axis), which shows whether genes have more or less common functional genetic variation relative to the genome wide expectation. A gene with a positive score has more common functional variation whereas a negative score shows the genes that are intolerant to functional variation. The scores are than assigned percentiles in decreasing order of scores, so that a higher score (genes having more common functional variation) is given a higher percentile. Each point shows a gene and the black line is the best fit line depicted for visualization purposes. The correlation between change in variability with age and RVI percentage is calculated using Spearman's correlation and the coefficient is given for each plot. The same analysis was repeated for the absolute value of the change in variability and also only using the significantly DV genes and the results are comparable with Spearman's

correlation coefficient ranging between -0.03 and 0.01 (graphs are not shown).

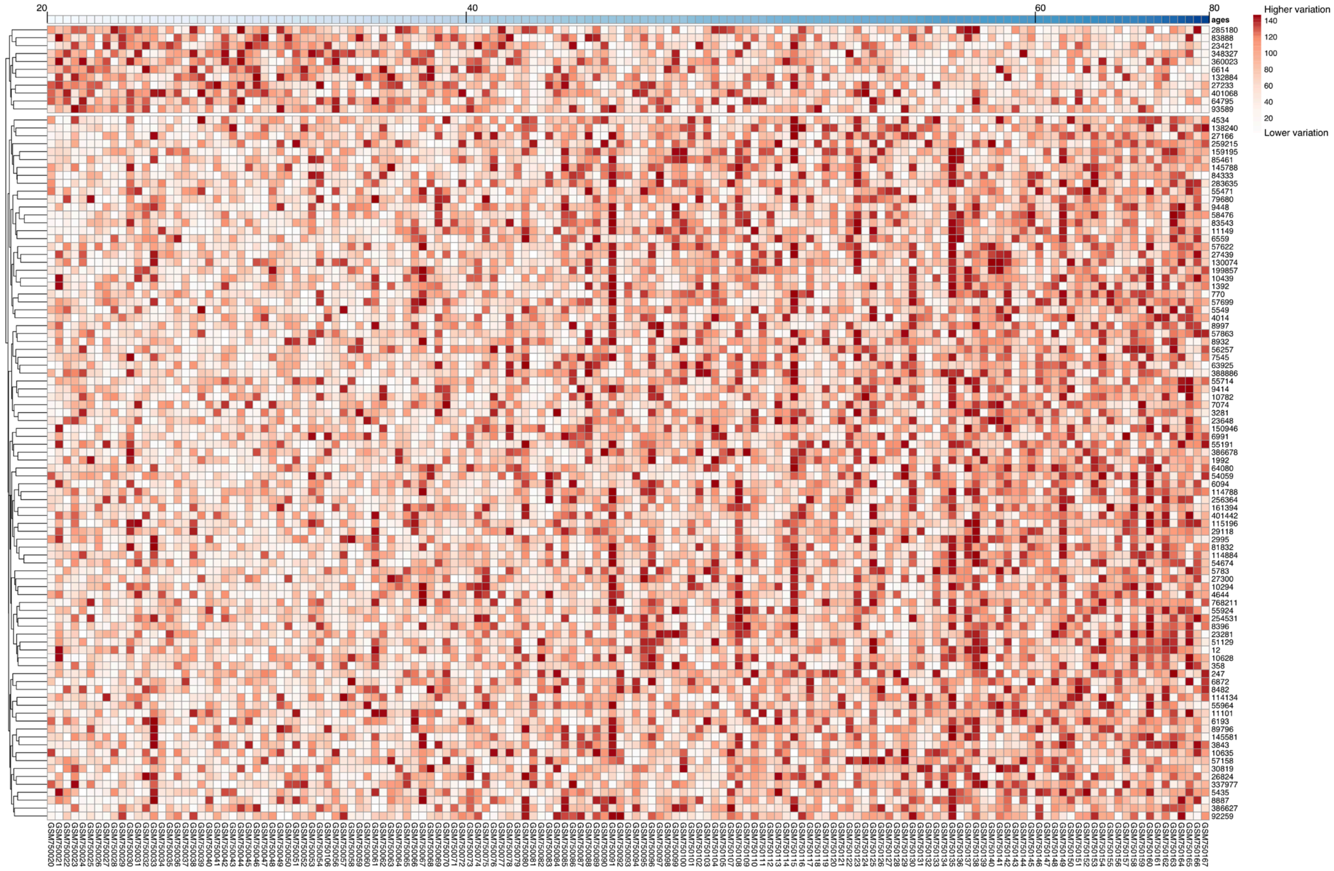

Supplemental Figure 14. A heatmap visualizing the variability for each individual (columns), and top 100 genes with the highest change in variability in either direction (rows) based on continuous approach and regression correction. The color shows the rank of individuals with respect to their variability for that particular gene. Darker colors show individuals with the highest variability for that particular gene. Genes are clustered using hierarchical clustering and individuals are ordered by age.

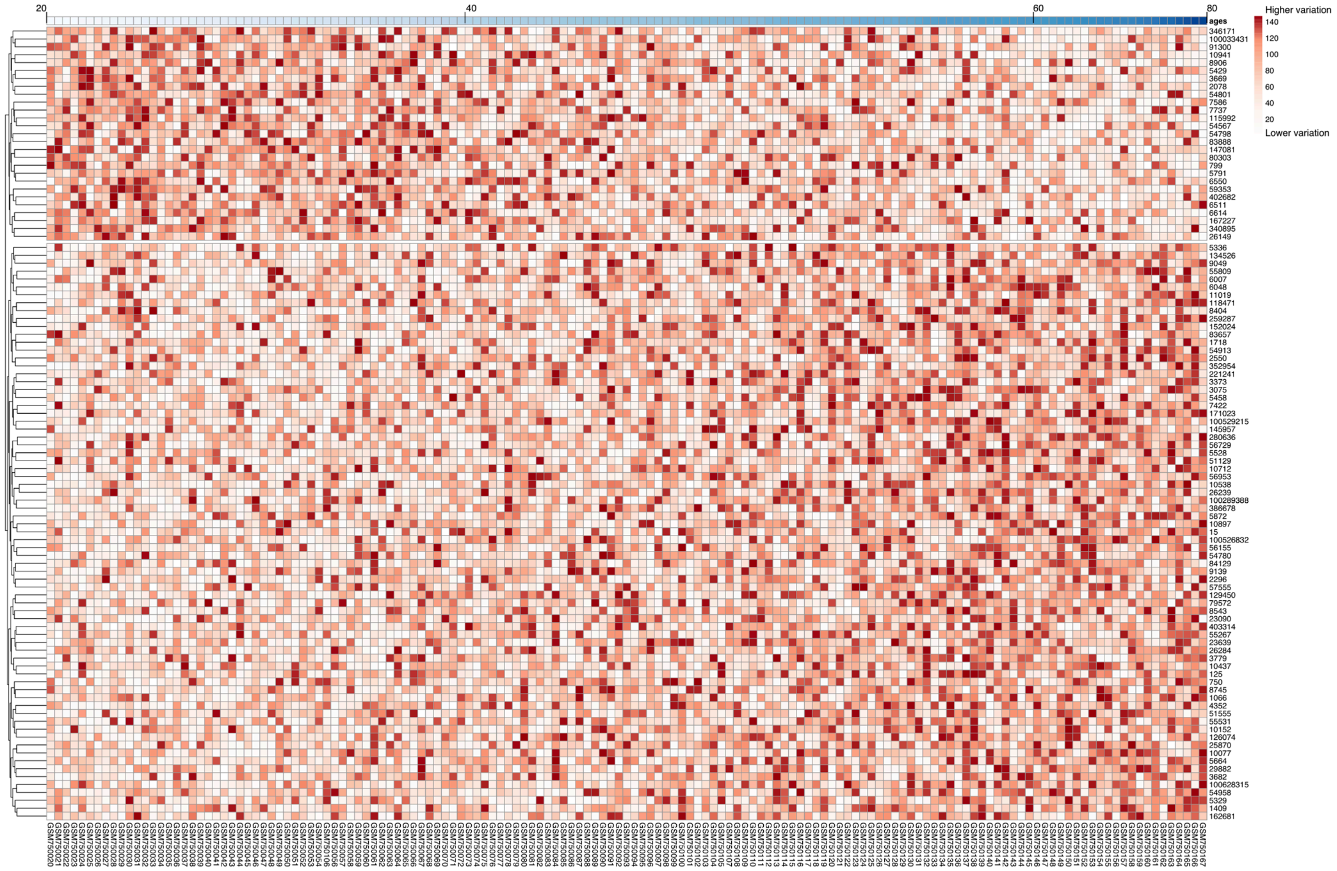

Supplemental Figure 15. Same as Supplemental Figure 14 but for continuous approach using SVA correction.
